# Editorial: Insights in microbial symbioses: 2021

**DOI:** 10.3389/fmicb.2022.1022893

**Published:** 2022-09-28

**Authors:** Zhiyong Li, Robert Czajkowski

**Affiliations:** ^1^State Key Laboratory of Microbial Metabolism, School of Life Sciences and Biotechnology, Shanghai Jiao Tong University, Shanghai, China; ^2^Laboratory of Biologically Active Compounds, Intercollegiate Faculty of Biotechnology University of Gdansk (UG) and Medical University of Gdansk (MUG), University of Gdańsk, Gdańsk, Poland

**Keywords:** microbial symbioses, rumen microbiota, seed endosymbionts, plant microbiota, animal microbiota

Frontiers in Microbiology has organized a series of Research Topics to emphasize the latest scientific innovations in different fields. This specific initiative, “*Insights in microbial symbioses: 2021*”, led by Dr. Zhiyong Li, Shanghai Jiao Tong University, China, and Professor Robert Czajkowski, University of Gdansk, Poland, is focused on new insights, novel developments, current challenges, and latest discoveries, recent advances and future perspectives in the field. The Research Topic aims to inspire, inform, and guide researchers in the field of microbial symbiosis research.

Six manuscripts were accepted for publication in this Frontiers Research Topic. Two papers investigate the symbiotic microbial community structure and function, for example, rumen microbiota of yaks (Han et al.) and endophytic fungi in *Alpinia zerumbet* seeds (Yan et al.). Two others examine the symbiotic bacterial function on plants (Yurgel et al.; Zhu et al.). The last two articles analyze the interaction between endobacteria and the fungi *Esteya vermicola* (Wang et al.), *Sirex noctilio* and *Amylostereum areolatum* (Fu et al.).

Rumen microbiota are closely linked to the feeding utilization and environmental adaptability of ruminants. However, knowledge about the impact of different extreme environments on the rumen microbiota of yaks is scarce. Han et al. compare the rumen fermentation parameters and microbiota of yaks from different altitude regions in Tibet, China, by gas chromatography and high-throughput sequencing. Principal coordinate analysis revealed significant differences in rumen microbial composition of yaks from different regions. Furthermore, the predicted function of rumen microbiota was found to differ between areas. These results reveal that regions located at different altitudes influence microbiota composition and the fermentation function of yaks' rumen, providing mechanistic insights on yak adaptation to high altitudes for improving the feeding efficiency of these animals in extreme regions.

Endophytic fungi act as seed endosymbionts, thereby playing a very crucial role in the growth and development of seeds. To investigate both the composition and diversity of endophytic fungi in *Alpinia zerumbet* seeds, high-throughput Illumina MiSeq sequencing was employed by Yan et al. The genera with the greatest abundance were *Cladosporium, Kodamaea, Hannaella, Mycothermus, Gibberella, Sarocladium*, and *Neopestalotiopsis*. Functional Guild (FUNGuild) analysis revealed that endophytic fungi belong to various types of saprotrophs and pathogens.

Bacterial inocula for improving plant growth and production are essential to sustainable agriculture. Yurgel et al. investigated the response of plant-associated microbiome to plant root colonization by exogenous bacterial endophytes in perennial crops. They monitored the effect of the inoculation of a single bacterial strain, an endophyte (RF67) isolated from *Vaccinium angustifolium* (wild blueberry) roots and characterized as *Rhizobium* spp. on the diversity, structure, and cooperation in plant-associated microbiome over 1 year. The findings presented by the authors suggest that while exogenous endophytes might have a short-term effect on the root microbiome structure and composition, they can boost cooperation between plant-growth-promoting endophytes.

Wild rice (*Oryza granulata*) is a natural source of abundant yet unknown endophytic fungal species. Zhu et al. isolated a new species *Pseudophialophora oryzae* sp. nov, from wild rice and proved that *P. oryzae* could promote rice growth and induce systemic disease resistance. Thus, they suggest that it can be further developed as a new biological control agent for agricultural applications, providing an innovative approach for the biocontrol of rice blast in the field.

Endosymbionts are found in the cytoplasm of nematophagous fungi *Esteya vermicola* from various geographical areas. Wang et al. sequenced the genome of endobacteria residing in *E. vermicola* to discover possible biological functions of these widespread endobacteria. The authors revealed that the endobacteria in *Esteya vermicola* contained multiple nematicidal subtilase/subtilisin encoding genes, indicating that it is likely that endobacteria could cooperate with the host to kill nematodes.

A strict relationship exists between *Sirex noctilio* and *Amylostereum areolatum*. In the investigation by Fu et al., real-time quantitative polymerase chain reaction (RT-qPCR) was used to measure gene expression in samples of *A. areolatum* at different growth stages and explore the essential genes and pathways involved in the growth and development of this symbiotic fungus. Furthermore, the stability of 10 candidate reference genes in symbiotic fungal samples was evaluated. Finally, p450, CYP, and γ-TUB were identified as suitable reference genes, laying a foundation for further gene expression experiments and understanding the symbiotic relationship and mechanism between *S. noctilio* and *A. areolatum*.

The articles in this Research Topic represent a wide range of areas in the field of microbial symbioses, including symbiotic microbial community structure and function, animal microbiome and plant microbiome, and the interaction between bacteria and fungi. Five of the six papers published are from China, indicating that microbial symbioses are becoming a field of intense research interest there.

## Author contributions

ZL wrote the editorial. RC revised the editorial. Both authors contributed to the article and approved the submitted version.

## Conflict of interest

The authors declare that the research was conducted in the absence of any commercial or financial relationships that could be construed as a potential conflict of interest.

## Publisher's note

All claims expressed in this article are solely those of the authors and do not necessarily represent those of their affiliated organizations, or those of the publisher, the editors and the reviewers. Any product that may be evaluated in this article, or claim that may be made by its manufacturer, is not guaranteed or endorsed by the publisher.

